# Monitoring of Cyclosporine A Blood Levels in Clinical Settings

**DOI:** 10.5812/numonthly.2701

**Published:** 2012-03-01

**Authors:** Mariusz Niemczyk

**Affiliations:** 1 Department of Immunology, Transplant Medicine and Internal Diseases, Medical University of Warsaw, Poland

**Keywords:** Cylclosporine A, Pediatric

Dear Editor,

I would like to comment on the paper by Beiraghdar et al. (1). C2 is regarded as a better indicator of cyclosporine A (CsA) exposure compared to C0 (2), this is also the case in pediatric patients (3). However, in clinical settings, C0 monitoring seems to be more reliable. It is connected to the fact that, CsA blood concentration is quite stable for between 10 and 12 hours after administration, and the collection of blood during that time allows valuable clinical results to be obtained. On the other hand, the peak concentration is obtained 2 hours after administration only in a limited group of patients, so called “good absorbers.” In delayed absorbers, C2 is not fully reliable(4). Limitations of C2 monitoring have also been proven in children (5). Additionally, monitoring CsA therapy with concomitant C0 and C2 observation is more expensive and connected to double stress and blood loss. Therefore, I would support the usage of C0 as the standard method of CsA therapy observation.

Beiraghdar et al. have found several correlations between CsA blood levels and clinical parameters. A negative correlation between CsA levels and serum creatinine, however, seems surprising. I would have expected completely the opposite relationship due to the well known nephrotoxic properties of CsA. Other correlations reported by the authors also seem difficult to explain. Unfortunately, the authors did not even try to account for their results and show which elements were causes, and which were effects. Many factors may be expected to impact on the pharmacokinetics of CsA. Therefore, they may only be correlated to C0 or C2 if the study group received the same dose of CsA per kilogram of body mass. As I understand, in the reported population, CsA doses were adjusted according to CsA blood levels, and varied in different patients. Therefore, C0 and C2 should not correlate with clinical parameters, as they depend mainly on CsA dosing. To obtain clinically relevant conclusions, clinical parameters should be correlated to CsA doses. Otherwise, the results would seem to have low significance for clinical practice. Finally, is it possible that these parameters will become important in clinical decision making? I do not think so. In my opinion, what will remain of key value is C0 and clinical experience.

## References

[1] Beiraghdar F, Rostami Z, Einollahi B (2011). Cyclosporine Through and 2 Hour Post Dose Monitoring and Its Contributing Factors Among Pediatric Kidney Recipients. Nephro-Urol Mon.

[2] Yoshimura N, Okamoto M, Akioka K, Kaihara S (2004). Optimization of the use of cyclosporine in renal transplantation.. Transplant Proc.

[3] Dunn SP (2003). Neoral monitoring 2 hours post-dose and the pediatric transplant patient.. Pediatr Transplant.

[4] Nashan B, Bock A, Bosmans JL, Budde K, Fijter H, Jaques B (2005). Use of Neoral C monitoring: a European consensus.. Transpl Int.

[5] Hmiel SP, Canter C, Shepherd R, Lassa-Claxton S, Nadler M (2007). Limitations of cyclosporine C2 monitoring in pediatric heart transplant recipients.. Pediatr Transplant.

